# Poemas de la Pradera: Transnational Identity Development Through YPAR and AI-Assisted Mayan Language Preservation

**DOI:** 10.3390/bs16060903

**Published:** 2026-06-02

**Authors:** Hector Palala Martinez, Edmund T. Hamann

**Affiliations:** Department of Teaching, Learning and Teacher Education, University of Nebraska–Lincoln, Lincoln, NE 68588, USA; hjpalala@gmail.com

**Keywords:** Mayan languages, transnational identity, youth participatory action research (YPAR), digital storytelling, language preservation, Indigenous youth, Nebraska

## Abstract

This study examines how transnational Mayan youth in rural Nebraska preserved and developed their linguistic heritage through the Kematzib’ Project while maintaining digital connections to Guatemala. Using Youth Participatory Action Research (YPAR) methodologies, K’iche’ and Q’anjob’al-speaking adolescents served as co-researchers, creating trilingual educational materials through poetry workshops and digital storytelling. Digital technologies facilitated both language preservation and transnational family connections, as participants utilized the same tools to create educational materials and maintain contact with their relatives in Guatemala. The collaborative creation of digital books that integrated *Popol Wuj* elements with contemporary transnational experiences helped students overcome linguistic shame, develop cultural pride, and build advocacy skills applicable across U.S. and Guatemalan contexts. This research illustrates how youth can activate transnational funds of knowledge while developing identities encompassing multiple national, cultural, and linguistic affiliations.

## 1. Introduction

The growing Mayan diaspora in the U.S. Midwest (Sittig & Gonzalez, 2016) presents unique challenges for youth identity development as young people navigate between Indigenous and Latiné heritages (a term used here to ensure gender-neutral representation) and contemporary American contexts (Sierk, 2016). This study examines transnational language and identity development among Mayan-heritage youth in rural Nebraska through YPAR, focusing on how digital connections to Guatemala and collaborative cultural production support complex identity formation within the Kematzib Project.

This study aligns with broader global priorities, particularly the United Nations Sustainable Development Goals concerning quality education and the reduction in inequalities for Indigenous and migrant populations. To explore these dynamics, this research utilizes Youth Participatory Action Research (YPAR), a methodological framework that positions young people as co-researchers, enabling them to generate knowledge about their own lives while pursuing transformative change (Cammarota & Fine, 2008). Furthermore, we examine how youth engage in translanguaging, the fluid, dynamic use of their full, multilingual repertoire, to navigate their environments and assert their identities.

At the time of this study/project, Wakefield Public Schools (WPS) in Nebraska enrolled just more than 500 students in a single K-12 building. In comparison to the state that has long predominantly enrolled European-descent White students, WPS was significantly more diverse: with 60% Hispanic/Latino students and 39% White. In turn, 30% were identified as English language learners (predominantly Spanish and Mayan-speaking multilingual Guatemalan immigrants), which was more than 3X higher than state average, and 60% were identified as economically disadvantaged students (as measured by free and reduced-price lunch eligibility), which also exceeded state average (Nebraska Department of Education, 2026). Community demographics were consistent with broader Guatemalan migration patterns to Nebraska’s agricultural communities, where families maintain strong transnational connections while establishing new lives (Griffith, 1995; Sittig & Gonzalez, 2016).

The Kematzib’ Project, named after the K’iche’ verb kematz’ib’ meaning “to write” or “to document”, offered an innovative approach to Mayan language preservation and development, integrating traditional cultural elements with contemporary digital technologies. As one participating student reflected, “At Wakefield School in Nebraska, we embarked on a journey to explore and revitalize our Mayan languages, Q’anjob’al and K’iche’.” The project was one of several concurrent initiatives pursued under the guidance of youth participatory action research (YPAR) that involved both authors, but the Kematzib’ Project was primarily driven by the first author who converted it into his dissertation project. The first author is a multilingual Guatemalan national who had previously taught in a dual-language elementary school in that country prior to securing a Fulbright to earn both a Masters and doctorate in the US. Multicultural education and instructional technology were twin interests of his graduate studies.

### 1.1. Global Language Endangerment and Mayan Languages

Of the more than languages spoken worldwide, over one-third are endangered, with an estimated one language disappearing every two weeks (Crystal, 2000; Sutherland, 2003). Globalization, colonial legacies, and the dominance of global languages accelerate this crisis (McCarty, 2002; Rigsby, 1987). Foundational studies have extensively documented the structural marginalization of Mayan linguistic communities and the broader decline of Indigenous languages worldwide (Crystal, 2000; McCarty, 2002; Sutherland, 2003). Scholars further estimate that between one-third and one-half of the world’s remaining languages may disappear within this century (Austin & Sallabank, 2011; Evans, 2010; Olko & Sallabank, 2021: Rehg & Campbell, 2018), with recent research demonstrating how linguistic ideologies, technological inequities, and uneven access to digital infrastructures intensify these vulnerabilities in the digital era (Rosillo-Rodes et al., 2023; Simons, 2019). Despite this growing body of scholarship, limited attention has been given to the behavioral agency of youth in diaspora contexts. This study addresses that gap by demonstrating how transnational youth strategically navigate—and at times circumvent—these structural constraints through digital tools and participatory practices to sustain, revitalize, and reinterpret their linguistic and cultural heritage. As less common and lower-status languages continue to be threatened, the hegemonic dominance of English and, to a lesser extent, Spanish means the numbers of users of these latter languages continues to grow (Moreno-Fernández & Otero, 2008).

For most of Guatemala’s history since Spanish arrival in the 1500s, Mayan language use and education in those languages have been discouraged, sometimes quite violently (Maxwell, 2020). For Mayan languages specifically, systemic marginalization in Guatemala, coupled with migration to the United States, exacerbates risks of language shift and loss (England, 2003; Foxen, 2007). In Nebraska, youth experience stigmatization of both Mayan language use and identity in schools and in public spaces, where speaking Indigenous languages marks them as outsiders (Jenner & Konkel, 2018). Yet accounts of domination and cruelty are incomplete without also acknowledging resistance. Mayan communities have historically maintained cultural resilience and developed sophisticated transnational networks to preserve heritage (Bastos, 2007). Still the active promotion/support of Central American indigenous languages as part of US schooling (as described later) was highly unusual from both contemporary and historic perspectives.

### 1.2. Transnational Identity and Sojourner Students

Transnational identity formation refers to inhabiting and negotiating multiple cultural contexts simultaneously (Levitt & Glick-Schiller, 2004). Vertovec’s (2007) concept of super-diversity explains how contemporary migrants combine varied cultural, legal, and linguistic affiliations to construct hybrid subjectivities. For Indigenous migrants, these identities are shaped further by sovereignty claims and colonial histories (Nájera & Maldonado, 2017; Quijano, 2000).

The interdisciplinary field of migration studies has long debated whether to consider those who are transnationally mobile as sojourners or settlers (i.e., continuously mobile or dislocatable versus stably settling in their new environments). The framework of sojourner students (Hamann, 2001; Hamann & Zúñiga, 2021; Zúñiga & Hamann, 2009) suggests that students’ enduring transnational ties, technology assisted communication, and participation in networks that include continuing transnational movement (even when a given individual does not) all mean declaring a full ‘settled’ status can be misleading or even hazardous.

The sojourner student framework is particularly relevant to Mayan youth in Nebraska. Students like those who were part of this initiative have often faced interrupted schooling, circular migration, and constant adaptation to new contexts, resulting in multiple but also constrained senses of belonging. Their lived experiences underscore the need for schools to incorporate transnational perspectives and develop culturally sustaining pedagogies. If constructivist educational principles suggest scaffolding on existing knowledge to build new understandings (Vygotsky, 1978) while related liberatory pedagogies urge ‘knowing the word to know the world’ (Freire, 1970), then accounting for students’ transnational pasts, presents, and futures all should factor in educational design and praxis.

## 2. YPAR with Indigenous and Immigrant Youth

Youth Participatory Action Research (YPAR) positions young people as co-researchers, enabling them to generate knowledge about their own lives while pursuing transformative change (Cammarota & Fine, 2008; Torre & Fine, 2006). Rooted in critical pedagogy (Freire, 1970) and the related liberatory adult mobilization framework labeled participatory action research (Fals-Borda, 1987), YPAR aligns with Indigenous methodologies that emphasize sovereignty, reciprocity, and community accountability (Caxaj, 2015; Tuck, 2009).

Research indicates that YPAR fosters identity development among immigrant youth by offering spaces for reflection on transnational experiences and systemic inequities (Caraballo et al., 2017), though researchers must actively work to prevent the unintentional reproduction of existing power structures during the process (Call-Cummings et al., 2019). For Indigenous immigrant youth specifically, YPAR offers opportunities to explore how colonial legacies intersect with contemporary migration experiences, while developing tools for advocacy and change.

Additionally, in Indigenous contexts, YPAR resonates with collective forms of knowledge-making, where cultural expression and self-determination are central to the research process. Anzaldúa’s (1987) notions of *nepantla* and borderlands consciousness provide frameworks for understanding how youth articulate hybrid identities while navigating and resisting marginalization. Youth can develop what she terms “mestiza consciousness” that embraces contradiction and multiplicity rather than seeking singular identity categories. Intersectionality and hybridity are more recent terms that highlight these premises.

### 2.1. Digital Transnationalism, AI, and Language Revitalization

Digital technologies enable migrants to maintain “connected migration” through ongoing communication with relatives abroad (Appadurai, 1996; Diminescu, 2008; Miller & Madianou, 2012). For Mayan youth, apps such as WhatsApp support not only social ties but also cultural and linguistic continuity. Digital storytelling, AI-assisted translation, and multimodal platforms extend these practices into educational and community contexts (Ajani et al., 2024).

In language revitalization, translanguaging and culturally sustaining pedagogy provide frameworks for sustaining linguistic diversity. García (2009) emphasizes bilingual education that affirms heritage languages, while Paris and Alim (2014) call for culturally sustaining pedagogies that actively support diversity. Building on this, Cenoz and Gorter (2017) have proposed translanguaging as a sustainable practice for minority languages.

Emerging scholarship on AI and Indigenous rights cautions against uncritical use of new tools, emphasizing community control, ethical safeguards, and cultural protocols (Carpenter & Tsykarev, 2020; Martín, 2023). Nonetheless, community initiatives, such as the Pixanixim Collective (2024) in Nebraska and ONIC (2016) in Colombia, demonstrate how Indigenous organizations integrate tradition with digital innovation.

### 2.2. Abya Yala as a Transnational Indigenous Framework

The Indigenous concept of *Abya Yala*, “land in its full maturity,” which comes from the Kuna language (Panama), offers a decolonized geography for situating Indigenous experiences across borders (Carcelen-Estrada, 2017; ONIC, 2016). By framing their identity within Abya Yala rather than within imposed nation-states, Indigenous community members can reaffirm their sovereignty and belonging, thereby transcending colonial divisions. For Mayan youth encountered in Nebraska, this framework supports reimagining transnational experiences as continuations of ancestral struggles and cultural persistence.

Three research questions guided the project.

How can Mayan youth use YPAR to navigate and articulate their transnational identities within the Kematzib framework?

What roles can digital connections to Guatemala and AI-assisted technologies play in identity development for diaspora youth?

How does collaborative cultural production (between high school students and their YPAR coordinator) augment student agency for affirmative identity development across national contexts?

## 3. Participants, Context, and Methods

### 3.1. Research Design and Positionality

This study employed Youth Participatory Action Research (YPAR) and Participatory Narrative Inquiry (PNI), centering Mayan adolescents as experts on their transnational lives. A central goal of YPAR is empowerment as it “teaches young people that conditions of injustice are produced, not natural” and are therefore “challengeable and thus changeable” (Cammarota & Fine, 2008, p. 2). Further, it centers the capacities of youth as “intellectual beings capable of engaging in the practice of critical investigation of community issues and the production of viable, usable knowledge” (Caraballo et al., 2017, p. 315).

As a Mayan-descendant researcher with experience as a teacher at bilingual (Kaqchikel and Spanish) Guatemalan public school, the first author adopted a co-learner stance, guided by Freire’s (1970) dialogical pedagogy. This shifted his role from that of a more positivist, traditional researcher to that of a facilitator. While the lead author functioned as a facilitator and provided training on digital tools, the participating youth acted as co-researchers who explicitly designed the peer interview questions, led the data collection during poetry workshops, and collaboratively identified the emerging themes during analysis.

The second author, a bilingual, white, tenured, professor of teacher education had co-led YPAR initiatives at this and other Nebraska sites and had introduced the first author to this setting. He was familiar with the environment and the participating youth, but not directly involved in the AI-deploying, trilingual efforts that were the generative sources of the exploration, development, and assertions of transnationalism, multilingualism, and youth identities described next. To ensure rigor, we applied Charmaz’s (2014) constructivist grounded theory principles, Creswell and Poth’s (2018) guidance on qualitative inquiry, and Loh’s (2013) criteria for trustworthiness. Credibility was supported through prolonged engagement, triangulation, peer debriefing, and member checks.

### 3.2. Participants and Community Context

The project was pursued with high school students in the small Nebraska town of Wakefield, which houses its entire K-12 500-student school enrollment in a single building, drew inspiration from broader Indigenous-led digital initiatives, such as the Pixanixim Collective, a Nebraska-based Maya community organization focused on cultural revitalization, and ONIC (National Indigenous Organization of Colombia), which advocates for Indigenous rights and autonomy in Latin America. These entities demonstrate how Indigenous organizations successfully integrate ancestral tradition with digital innovation.

Three Guatemalan Mayan youth served as co-researchers. Using the names they selected, they were: Ixbalanqué (17, Q’anjob’al speaker), Vucub-Hunahpu (17, K’iche’ speaker), and Hun-Hunahpu (16, K’iche’ speaker). All had migrated to Wakefield within the last three years and maintained digital ties to Guatemala. Thus, various geographic locales in Guatemala together with Wakefield, Nebraska became parts of plural-geography “transnational community” (Guerra, 1998). Wakefield, although small, was significantly transnationally connected and shaped by labor migration dynamics of the meatpacking industry (Griffith, 1995; Sittig & Gonzalez, 2016).

## 4. The Kematzib’s Project

The Kematzib project unfolded through three interconnected phases, positioning youth as digital storytellers and cultural knowledge holders. The first phase focused on identity mapping and question development, where the youth mapped their migration experiences and developed peer interview questions that explored language use and identity formation across transnational spaces. The second phase involved collaborative data collection with AI integration, as co-researchers conducted interviews, facilitated poetry workshops, and engaged in digital documentation processes. During this phase, they integrated AI tools such as ChatGPT-4o, DALL·E 2, and Gemini 1.5 for translation support and visual storytelling, using these technologies to amplify their voices and create multilingual narratives that honored their linguistic diversity.

The final phase centered on cultural production and analysis, where co-researchers created trilingual poetry and AI-assisted digital books that served as both data and creative expression. Through this process, they analyzed emerging themes from their collective work. They presented their findings publicly (including at a multi-high school ‘YPAR Summit’), transforming their transnational experiences into digital stories that could reach both local and transnational audiences. This methodology honored Indigenous storytelling traditions while embracing contemporary digital tools and allowed youth to incorporate family and heritage knowledge while maintaining agency over their narrative representations.

### 4.1. Data Sources

The research drew from multiple interconnected data sources to capture the complexity of youth transnational experiences. To clarify the scale of inquiry, the three focal youth participants engaged in two semi-structured interviews each, resulting in six total interviews (each lasting 60–90 min). Table 1 summarizes the comprehensive data collection methods utilized in this study.

### 4.2. Data Analysis

The analysis was guided by constructivist grounded theory, which views meaning as co-constructed between participants and researchers (Charmaz, 2014). Consistent with Youth Participatory Action Research, analysis moved beyond conventional text-based coding to include creative praxis (Cammarota & Fine, 2008). This allowed the multilingual dimensions of Q’anjob’al, K’iche’, Spanish, and English to remain central throughout interpretation. Data sources included interview transcripts, poetry workshops, focus groups, field observations, and digital artifacts. Initial open coding identified recurring themes related to migration, language, identity, family, and transnational belonging. Emerging codes were then revisited collaboratively with the youth co-researchers, allowing categories and themes to develop through dialog, reflection, and shared interpretation.

Poetry, drawing, music, and digital storytelling were treated as primary analytic tools rather than supplementary materials, enabling participants to express emotional, cultural, and linguistic experiences that might not fully emerge through transcripts alone (Roberts, 2013). This arts-based approach supported embodied meaning-making and interpretation grounded in participants’ lived experiences (Moraga & Anzaldúa, 2022).

To maintain participant agency and cultural accuracy, youth co-researchers actively participated in theme development, interpretation, and member-checking throughout the analytical process. Through this collaborative and embodied approach, data were understood not simply as information to be extracted, but as collective stories to be interpreted, felt, and composed in community (Charmaz, 2014; Roberts, 2013).

## 5. Theme 1: Language Maintenance Through Digital Practice

### 5.1. Digital Bridges: AI-Assisted Connections to Homeland

Family communication via WhatsApp became a site of and means for cultural transmission:

*“I use WhatsApp to connect with my grandparents, cousins, and uncles in Guatemala. We speak K’iche’ during these calls.”*

—Vucub-Hunahpu, age 17

AI tools supported representation without replacing cultural authority. Hun-Hunahpu (age 16) described sharing a trilingual poem with her grandmother: “She cried and said she was proud I had not forgotten our words. Using AI helped me create a digital book about my story that I could share with my Nebraska classmates and my family in Guatemala.” Unpacking this example a little more, Hun-Hunahpu would not have described herself as fully adept with K’iche and had never previously used it in a US academic environment, but AI helped her generate text in translation that she then curated—Did it sound right to her and classmates who knew K’iche? She then sought (and received) full affirmation for when she shared it with her grandmother. What began tentatively was affirmed with the enthusiastic response of a high-value social contact (i.e., her grandmother). We could add that the shared artifact—the poem—also displayed to her grandmother her growing competence and confidence with English and Spanish (which her grandmother did not need to be able to fully comprehend to nonetheless feel pride in her granddaughter’s apparent competence with it.

### 5.2. Poetry as Identity Work: Trilingual Cultural Production

Initially, participants hesitated to use Indigenous languages in public due to stigma and their own unfamiliarity with using them for/with academic tasks. So, poetry workshops became spaces of identity assertion and reclamation.

Ixbalanqué’s trilingual poem “Awal yet chi wab’—When I feel I have no strength left”:

*K’iche’ (original)*

*Awal yet chi wab’ que k’am xa wip, ka chi na on tek’ que eb’ in’ mam y txutx…*

*Spanish*

*Cuando siento que ya no tengo fuerzas, recuerdo que mis padres…*

*English*

*When I feel like I have no strength left, I remember that my parents have never given up and have worked tirelessly for my siblings and me every day…*

Ixbalanqué explained:

“I wrote first in Q’anjob’al because that is the language of my heart when I think about my family. However, I wanted everyone to understand—my teachers, my friends, my family in Guatemala—so I made it in all three languages.”

**Figure 1:**
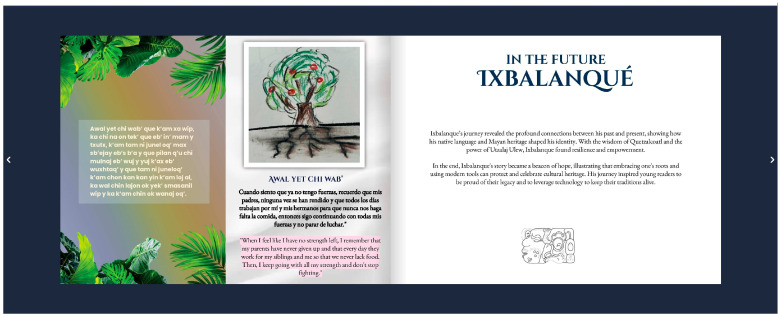
Ixbalanqué’s Poem “Awal yet chi wab’ digitalized.

Figure 1 helps illustrate how discrete project creations actually functioned as complementary components. Ixbalanqué’s drawing was not separate from his trilingual text production, and the assistance of the technology made it relatively easy to give the whole effort a professional (appearing) production gloss.

## 6. Theme 2: Transnational Identity Negotiation

### 6.1. “We Are Mayas in Nebraska”: Articulating Transnational Belonging

Youth rejected either/or identities, instead coining the phrase “We are Mayas in Nebraska.” This was more than ‘both/and’ work as assertions of indigenous identity had overlays of both Guatemalan and US contexts. To the former, asserting being Mayan was an act of resistance and survival, given the centuries of violence and subordination directed until very recently at indigenous identities in Guatemala. During workshops and focus groups, participants fluidly utilized K’iche’, Q’anjob’al, Spanish, and English. Crucially, interacting in Spanish to claim solidarity with fellow Guatemalan newcomer peers meant coopting a language that was hegemonically dominant and historically colonial in Guatemala. In this rural Midwestern space, however, the youth transformed Spanish into a strategic vehicle for self-asserted broader solidarities, using it not for assimilation, but to build a unified Indigenous-migrant coalition. This finding reflects the participatory nature of YPAR, as youth actively shaped interpretations of their transnational experiences through collaborative, multilingual analysis.

“Life has thrown many challenges my way, but I remain determined to stay connected to my roots. I struggle not to lose Q’anjob’al, but with my family and community, I feel more comfortable and value speaking our language. It is a constant balance, trying to hold onto who I am while fitting into this new place.”

—Ixbalanqué, age 17

**Figure 2:**
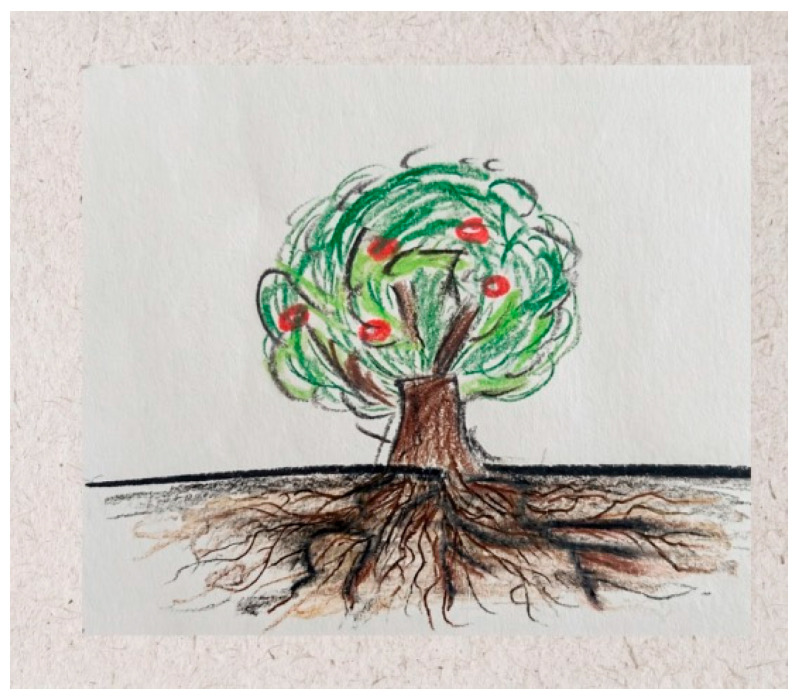
Ixbalanqué’s drawing of a fruit tree.

YPAR participants were encouraged to explore various modalities for developing and expressing their knowledge. While the tree depicted in Figure 2 may at first seem relatively rudimentary, there is no mistaking the depiction’s emphasis on roots, which Ixbalanqué said represented the Q’anjob’al Mayan language. From their themes of ‘hidden depth’, ‘grounding’ or ‘foundation,’ and intertwined substance that manifests in different forms (fruit, foliage, trunk, and roots) but as part of the same whole. Poetry workshops and digital projects helped transform shame into pride, reframing cultural and linguistic heritages as competencies and strengths.

### 6.2. Visual and Symbolic Representations of Identity

**Figure 3:**
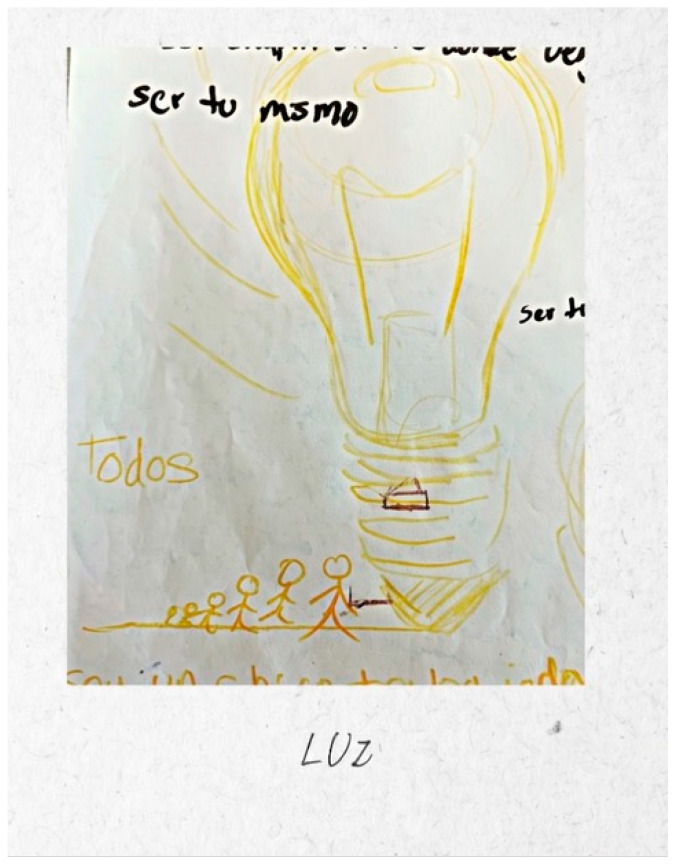
Vucub-Hunahpu’s drawing a light bulb representing K’iche’ Language.

Other participants also created symbolic images to express identity (like Ixbalanqué’s tree). Regarding an image of light bulb (See Figure 3), Vucub-Hunahpu explained: “I drew a light bulb that illuminates my life and my family’s. That is what the K’iche’ language means to me—it is being myself in the world.”

**Figure 4:**
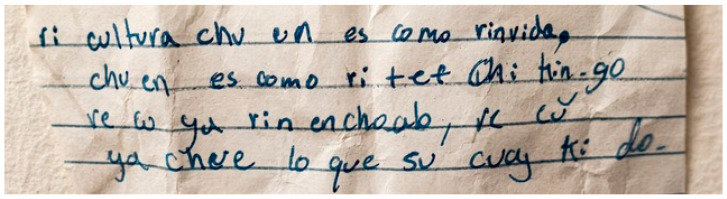
Poem in (K’iche’): Themes included the Mayan language as the mother’s womb, a secret language, past and present, and Mayan pride.

He later added: “I wrote a poem in K’iche’ about what our language means to me… it is like being inside my mother’s womb, giving me life and energy to survive.” An examination of the poem (Figure 4) shows a translanguaging text that draws from both Spanish and K’iche without a need to parse or translate one part of it or the other (as Vucub-Hunahpu could ‘read’ and generate both).

### 6.3. Trilingual Expressions of Belonging Across Borders

Again writing poetically, Hun-Hunahpu articulated hybrid identities that transcended geography:

*“I am from Guatemala, but I was born in Nebraska.*

*I have never traveled to Guatemala, but I know its traditions, its music, and its people.*

*I am like the moon, which at night reminds me of Guatemala, and the sun,*

*because the day reminds me of Nebraska.”*

Her reflections reveal transnational belongings that are rooted in both lived experience and cultural imagination. Here too, they are not ‘either/or’, but ‘both/and’. One can imagine the joy of a (Guatemala-based) grandmother reading/seeing assertions of her granddaughter’s Guatemalan-ness and the way this project, among its many other accomplishments, can be a vehicle for receiving familial affection and affirmation across distance.

### 6.4. Ethical Considerations

Ethical considerations were central to this project, particularly given the transnational and sometimes vulnerable contexts of the youth. Informed consent was secured, emphasizing the avoidance of harm and deep respect for cultural diversity. Youth asserted absolute authority over their representations, echoing Indigenous scholars’ emphasis on data sovereignty and community control. Their insistence that AI serve community goals reflects broader critiques of technological appropriation, ensuring the research process remained a culturally sustaining and empowering experience.

## 7. Discussion

The Kematzib’ Project demonstrates how Youth Participatory Action Research (YPAR), grounded in Freire’s (1970) pedagogy of critical consciousness, enables Guatemalan-background Indigenous youth to become co-researchers and cultural producers. This finding echoes the transformative role of YPAR in enhancing agency among marginalized youth (Cammarota & Fine, 2008; Caraballo et al., 2017; Torre & Fine, 2006). These results align with culturally sustaining pedagogy (Paris & Alim, 2014) and bilingual education frameworks (García, 2009), but importantly, they extend these theoretical models by demonstrating how AI-assisted tools can actively support the development of multilingual identity in transnational Indigenous contexts. Rather than being positioned as objects of study, participants used AI (ChatGPT, DALL-E, and Gemini) not as replacements for cultural knowledge, but as amplifiers to create knowledge frameworks that validated their hybrid identities and maintained their cultural authority.

Here digital technologies facilitated “connected migration” (Diminescu, 2008), as WhatsApp and other platforms became additional learning and communication spaces for language maintenance and cultural exchange. Such practices reflect Indigenous-led digital initiatives, such as the Pixanixim Collective (2024) in Nebraska and ONIC (2016) in Latin America, which demonstrate how communities sustain cultural continuity and social connection through digital means.

Ethical considerations were central to this project. Youth asserted authority over their representations, echoing Indigenous scholars’ emphasis on data sovereignty and community control (Carpenter & Tsykarev, 2020; Martín, 2023). Their insistence that AI serve community goals reflects broader critiques of technological appropriation and coloniality (Nájera & Maldonado, 2017; Quijano, 2000). This project was more in keeping with Camacho and Zevallos’ (2020) assertion that digital technologies can and must play a role in the support and preservation of lesser-used and endangered languages.

Poetry and digital storytelling transformed linguistic shame into pride, resonating with Anzaldúa’s (1987) borderlands consciousness and Addington et al.’s (2020) framing of digital decolonization. This mirrors Ak’abal’s (2019) blending of ancestral imagery with modern themes and also aligns with Olko and Sallabank’s (2021) argument that revitalization must produce new creative forms rather than only transmit tradition. Through trilingual poems and AI-enhanced books, participants enacted cultural sovereignty, reinforcing that language revitalization is also about generating innovative forms of expression (Cahnmann-Taylor, 2009, 2013).

Structural conditions of migration shape these identity processes. Rural Nebraska communities, sustained by meatpacking labor, create contexts of precarity and racialization (Griffith, 1995; Sittig & Gonzalez, 2016). Schools often become the primary sites where youth negotiate their identities as sojourner students (Hamann, 2001; Hamann & Zúñiga, 2021; Zúñiga & Hamann, 2009). The Kematzib’ Project reflects broader Indigenous frameworks of *Abya Yala* (Carcelen-Estrada, 2017; ONIC, 2016), situating youth identity work as part of hemispheric struggles for cultural sovereignty and continuity.

This research demonstrates that youth-centered inquiry through YPAR can be a powerful framework for centering transnational Indigenous youth voices in identity development processes (Cammarota & Fine, 2008). Educational institutions should adopt responsible AI integration that promotes multilingual expression, guided by community protocols and Indigenous values (Carpenter & Tsykarev, 2020; Martín, 2023). Rather than viewing multilingualism as a deficit, schools should sustain translanguaging as a pedagogical asset that honors and develops students’ full linguistic repertoire (Cenoz & Gorter, 2017; García, 2009). Moving beyond mere accommodation, education should adopt culturally sustaining pedagogy that actively maintains cultural pride and resilience (Paris & Alim, 2014).

Policy frameworks must recognize Indigenous languages as critical components of youth well-being and identity formation (Camacho & Zevallos, 2020; Whalen et al., 2016). This recognition should translate into funding for community-led, technology-enhanced language revitalization initiatives that honor Indigenous self-determination (UNESCO, 2022). Education and migration policies need to acknowledge the complex realities of transnational Indigenous identities, moving beyond singular national frameworks to embrace the borderlands experiences of youth who maintain connections across multiple territories (Hamann, 2001; Hamann & Zúñiga, 2021). Supporting cross-border collaborations, such as those led by the Pixanixim Collective (2024), can strengthen transnational Indigenous networks and cultural continuity.

The integration of AI technologies in Indigenous research and education must prioritize community consent and control throughout the design and implementation process (Martín, 2023). This includes embedding Indigenous governance structures within technology projects to prevent cultural appropriation and ensure that technological tools serve community-defined goals. Ensuring equity and accessibility remain paramount, particularly for marginalized learners who may face multiple barriers to technological engagement (Mayeux, 2024). Future technological developments should be guided by Indigenous data sovereignty principles that maintain community ownership over cultural knowledge and digital representations, ensuring the preservation of cultural heritage.

### 7.1. Limitations

This study only involved three youth co-researchers in one Nebraska school. While their depth of engagement offered rich insights, only limited generalizations are possible (Creswell & Poth, 2018), like that Indigenous transnational youth *can* find AI to be helpful support for trilingual, multi-modal identity expression. AI platforms often carry biases against underrepresented languages (Rosillo-Rodes et al., 2023), which can constrain the expression of creativity among young people. That was partially compensated for here by turning to peers (locally) and extended family (transnationally) to double-check AI-generated language, but such strategies are both cumbersome and imperfect.

### 7.2. Conclusions

The Kematzib Project showcased how Mayan youth in diaspora settings utilized ancestral knowledge, poetry, and AI technologies to affirm their identities across borders. Through YPAR, adolescents transformed linguistic hesitation and secrecy into cultural pride, creating trilingual poetry and digital books bridging Nebraska and Guatemala. Findings illustrate that Indigenous youth strategically integrated AI and digital tools to maintain cultural integrity while expanding expressive possibilities. Their agency challenges deficit assimilation models and affirms Indigenous innovation in language revitalization.

By situating this work within *Abya Yala* (Carcelen-Estrada, 2017; ONIC, 2016), borderlands consciousness (Anzaldúa, 1987), and critical pedagogy (Fals-Borda, 1987; Freire, 1970), the study shows that Indigenous belonging transcends national borders. As the International Decade of Indigenous Languages advances (UNESCO, 2022), projects like Kematzib provide models for ethically integrating technology into language revitalization, sustaining futures where youth voices remain central.

## Figures and Tables

**Table 1:** Data Collection Summary.

Method	Quantity	Participant Involvement/Details
Semi-Structured Interviews	6 sessions (60–90 min)	2 interviews per focal youth; co-facilitated and analyzed by youth
Poetry Workshops	12 sessions (~40 min each)	Youth-led creative spaces for trilingual expression and meaning-making
Focus Groups	6 sessions	Collective reflection on transnational experiences
Formal Field Observations	40 h	Systematic observation of contextual dynamics by the lead author
Digital Artifacts	Multiple	Trilingual poems, AI-assisted visual books, WhatsApp screenshots

## Data Availability

The original contributions presented in this study are included in the article. Further inquiries can be directed to the corresponding author.
